# Involvement of Oxytocin Receptor/Erk/MAPK Signaling in the mPFC in Early Life Stress-Induced Autistic-Like Behaviors

**DOI:** 10.3389/fcell.2020.564485

**Published:** 2020-10-02

**Authors:** Jinbao Wei, Le Ma, Peijun Ju, Beibei Yang, Yong-Xiang Wang, Jinghong Chen

**Affiliations:** ^1^Shanghai Key Laboratory of Psychotic Disorders, Shanghai Mental Health Center, School of Medicine, Shanghai Jiao Tong University, Shanghai, China; ^2^King’s Lab, School of Pharmacy, Shanghai Jiao Tong University, Shanghai, China; ^3^Hubei Key Laboratory of Wudang Local Chinese Medicine Research, Institute of Wudang Traditional Chinese Medicine, Taihe Hospital, Hubei University of Medicine, Shiyan, China

**Keywords:** early life stress, neonatal maternal separation, oxytocin receptor, Erk/MAPK signaling, H3K4Me3

## Abstract

The neonatal or infant period is a critical stage for the development of brain neuroplasticity. Early life stresses in the neonatal period, including neonatal maternal separation (NMS), have adverse effects on an increased risk of psychiatric disorders in juveniles and adults. However, the underlying molecular mechanisms are not largely understood. Here, we found that juvenile rats subjected to 4 h daily NMS during postnatal days 1 to 20 exhibited autistic-like behavioral deficits without impairments in learning and memory functions. Molecular mechanism studies showed that oxytocin receptor (OXTR) in the medial prefrontal cortex of NMS rats was evidently downregulated when compared with control pups, especially in neurons. Erk/MAPK signaling, the downstream coupling signaling of OTXR, was also inhibited in NMS juvenile rats. Treatment with oxytocin could relieve NMS-induced social deficit behaviors and activated phosphorylation of Erk/MAPK signaling. Furthermore, medication with the inhibitor of H3K4 demethylase alleviated the abnormal behaviors in NMS rats and increased the expression of OXTR in the medial prefrontal cortex, which showed an epigenetic mechanism underlying social deficits induced by NMS. Taken together, these findings identified a molecular mechanism by which disruptions of mother–infant interactions influenced later displays of typical social behaviors and suggested the potential for NMS-driven epigenetic tuning of OXTR expression.

## Introduction

Early life, including early childhood, neonatal, or even infant period, is a time of significant brain development and, therefore, a time when early social experiences influence the development of the central nervous system and expression of behaviors in subsequent adolescence and adulthood (Cushing and Kramer, 2005; Anda et al., 2006; Tian et al., 2018). Emerging pieces of evidence suggest that adversities during early life may contribute to a greater risk of developing mental disorders such as autism spectrum disorder (ASD), anxiety, and depression (Chapman et al., 2004; Carr et al., 2013; Flouri et al., 2020). Thus, early life stress (ELS) events are a cause or a predisposing factor for psychiatric diseases later in life.

Daily separation of newborns from their mothers for some hours during the neonatal period known as neonatal maternal separation (NMS), a model of ELS that leads to a disruption of maternal care, may have important implications for offspring neurodevelopment and disease risk (Carlyle et al., 2012; Loewy et al., 2019). As proved in animal studies, NMS led to anxiety-like behavior, accompanied by increased activity of hypothalamic–pituitary–adrenal (HPA) axis in rats (Daniels et al., 2004; Odeon and Acosta, 2019), and impaired localized *de novo* protein translation at synaptic connections in the rat hippocampus (Ahmad et al., 2018). Moreover, ELSs may be involved in deficits in social recognition (Kambali et al., 2019). Our behavioral results indicated that maternally separated rats during adolescence showed impaired social novelty preference for strange rats and increased preference for marble-burying behavior. These impairments were typical performances of autistic-like behavioral deficits that were characterized by the core symptoms of impaired social recognition, language communication failure, and stereotyped behaviors (Ferguson et al., 2001). Recent studies have identified that oxytocin (OT) and the oxytocin receptor (OXTR) exert important roles in the regulation of complex social behaviors such as maternal behavior, mating, aggression, attachment, sexual behavior, as well as in psychiatric disorders characterized by social deficits (Ferguson et al., 2001; Bakermans-Kranenburg and van Ijzendoorn, 2014; Hoge et al., 2019). The neuropeptide OT produced by the supraoptic nucleus and paraventricular nucleus of the hypothalamus in the brain, and various peripheral organs and membranes (kidney, amnion, heart, uterus, etc.), has been revealed as a profound anxiolytic and anti-stress factor of the brain with activation Raf/MEK/Erk/CREB (Erk/MAPK) signaling, an extracellular signal-regulated kinase pathway, besides its reproductive and prosocial effects (Zhong et al., 2003; Aoki et al., 2014; Neumann and Slattery, 2016). Due to its functions on the improvement of social recognition, intranasal OT has been under clinical studies for use in the treatment of adults and juveniles with ASD (Anagnostou et al., 2012; Penagarikano, 2015). Furthermore, OXTR variants were involved in children subjected to emotional neglect (Womersley et al., 2019). Deficits by knockout of OXTR or OT could lead to pervasive social deficits in mice (Takayanagi et al., 2005). These findings suggest that OT/OXTR system is linked to psychiatric disorders associated with social deficits and may be a potential therapeutic target for social deficits in ASD. Early life adversity events exert long-lasting influences on the brain via epigenetic regulatory mechanisms, making an individual susceptible to later psychiatric disorders (Unternaehrer et al., 2015; Seo et al., 2016). Early life experiences could cause epigenetic variability of OXTR in the brain implicated in social behavior (Kraaijenvanger et al., 2019; Tops et al., 2019).

We found that OXTR in juvenile NMS rats was significantly downregulated than control littermate pups, accompanied by phosphorylation inhibition of its downstream Erk/MAPK signaling in the medial prefrontal cortex (mPFC), an important brain region that plays an essential role in mediating social cognition (Amodio and Frith, 2006). Due to the critical role of the OT/OXTR system in the regulation of social behaviors, we hypothesized that repressed OXTR/Erk/MAPK signaling was implicated in NMS-induced social deficit. Our results showed that OT treatment relieved the social deficit of NMS rats with activation of Erk/MAPK signaling, demonstrating that the OT signaling was involved in social deficit in NMS rats. OXTR expression was regulated via tri-methylation of lysine 4 on histone H3 (H3K4me3), a marker for transcriptional activation that could provide support for the increase in OXTR messenger RNA (mRNA) level (Greer and Shi, 2012; Miller, 2016). H3K4me3 level was regulated by histone demethylases (HDMs) and methyltransferases (HMTs), which were widely expressed in different functional brain regions including PFC, hippocampus, hypothalamus, etc. (Kouzarides, 2007). KDM5B, a critical HDM, served as the main regulatory factor of H3K4me3; knockdown of which by small interfering RNA could increase OXTR expression via upregulating H3K4me3 (Miller, 2016). It was found that NMS caused a decrease of H3K4me3 level in the mPFC. Consequently, our studies further investigated whether NMS stress changed OXTR expression through epigenetic modulation of the histone methylation pathway. These findings demonstrated that NMS-induced epigenetic changes at OXTR might be predictive adaptive outcomes that facilitate behavioral pathology.

## Materials and Methods

### Animals

Timed-pregnant (15 days pregnant) Sprague-Dawley female rats were purchased from Shanghai Leigen Biotechnology Co., Ltd. All dams were housed in standard laboratory housing rooms with controlled temperature (23 ± 2°C) and humidity (25–70%) under a 12/12-h light/dark cycle (lights on at 7:30 a.m.). They were given standard food and drinking water *ad libitum*. Pregnant females were individually housed until delivery. In total, 94 male offsprings (assigned into four batches assays) were used in this study. All of the animal experiments were performed according to international guidelines and approved by the Experimental Animal Committee of Shanghai Jiao Tong University School of Medicine.

### Neonatal Maternal Separation Procedure

The birth date was considered as postnatal day 0 (PND 0). When pups were born, they were subjected to the NMS paradigm at PND 1. The NMS paradigm, similar to previous protocols (Amini-Khoei et al., 2015; Amiri et al., 2016), was used with slight modifications. The male offsprings were separated from their mothers for 4 h daily during PND 9–15 for a short-term separation or PND 1–20 for a long-term separation, beginning at 10:00 a.m., and were returned to their mothers after the 4-h separation period. During the separation period, the NMS offsprings were placed in separate compartments, and no nutritional supplements were provided. The offsprings were weaned on PND 21 and housed in groups (four rats per cage) until experiment day PND 38. The control littermates were undisturbed, weaned on PND 21, and grouped in cages (*n* = 4) till PND 38 for behavioral tests. All animals were handled for several times before the behavioral tests and acclimated to the behavior testing room 1 h to reduce stress and anxiety.

### Drug Administration

This study of the pharmacological intervention was carried out in two different parts. For the first part, to investigate the effects of OT administration on NMS juvenile rats, OT (Sandoz, United Kingdom) was dissolved in sterile 0.9% saline and intraperitoneally (i.p.) (1 mg/kg, 30 min before every behavioral test, i.p.) administered to the animals (NMS + OT group, nine males). The control littermates (CONT group, nine males) and other NMS juvenile rats (NMS group, nine males) were treated with an equal volume of 0.9% saline (30 min before every behavioral test, i.p.).

For the second part, the HDMs inhibitor As-8351 (Sigma131 Aldrich, Germany) was dissolved in 100% dimethyl sulfoxide and diluted to a 25% dimethyl sulfoxide solution (vehicle) with 0.9% saline for i.p. injection of 2 mg/kg. As-8351 was daily administered to the animals (NMS + AS-8351 group, nine males) on PND 21–35. Correspondingly, the CONT group (nine males) and NMS group rats (nine males) were received with an equal volume of the vehicle on PND 21–35.

### Behavioral Testing

#### Three-Chamber Sociability and Social Novelty

The three-chamber test is used to assess cognition in the form of general sociability and interest in social novelty in rodent models of psychiatric disorders (Qin et al., 2018). Thus, we used the three-chamber test to identify the effects of NMS on deficits in sociability and/or social novelty in juvenile female rats. Briefly, an apparatus (L: 60 cm, W: 40 cm, H: 22 cm, equipped with EthoVision XT 10 software package, Noldus Information Technology; Leesburg, VA, United States) containing three chambers with openings between the chambers allowing rats to access into side chambers was used. During the habituation, two empty capsules (an inverted pencil cup, D: 8 cm, H: 15 cm, placed in the center area) were placed at side chambers, and the subject was allowed to explore for 10 min. After habituation to the apparatus with two empty capsules, the subject was returned to the home cage. The durations staying in the side chambers were recorded. Those spending longer than 50 s in one side chamber than the other side were excluded in the following tests. For the sociability test, then, a never-before-met social rat (age- and sex-matched rat of the same strain, stranger rat 1, S1) was placed under one of the empty capsules, and the subject was placed in the center chamber and free to investigate the apparatus for 10 min. After the sociability measurement, the test subject was returned to the home cage. It took a 5-min interval for the next measurement. For the social novelty test, a new never-before-met social rat (stranger rat 2, S2) was placed under the other capsule, and the previous social rat now served as the familiar rat (S1). The subject then was placed again in the center chamber and free to investigate the apparatus for 10 min with a choice between the first, now-familiar, social rat (stranger 1) in one side chamber and a second unfamiliar rat (stranger 2) in the other side chamber. During the sociability and social novelty tests, the time spent sniffing each capsule, the time spent in each chamber, and the numbers of entries into each chamber were recorded and analyzed. After the tests, the two social rats and subjects were returned to their home cages. The chambers and capsules were cleaned with 75% ethanol after the tests.

#### Social Approach

As a more sensitive measure for sociability, we also used the social approach test to investigate the variation of social behavior in juvenile rats. After, the subject was placed in an apparatus (L: 58.5 cm, W: 58.5 cm, H: 58.5 cm) containing an empty capsule (an inverted pencil cup, D: 8 cm, H: 15 cm, placed in the center area) to explore for 10 min, then was returned to the home cage (Qin et al., 2018; Sharon et al., 2019). A never-before-met social rat (age- and sex-matched rat) was placed under the capsule. Subsequently, the subject was placed back into the apparatus and allowed to interact with the stimulus rat for 10 min, while the interaction time was recorded using an overhead video camera and tracked using the EthoVision XT 10 software package (Noldus Information Technology; Leesburg, VA, United States).

#### Novel Object Recognition

Rats show a preference to interact with a novel than with a familiar object. Novel object recognition test has been used to study learning and memory for behavioral and psychiatric disorders (Bevins and Besheer, 2006). To an apparatus (L: 58.5 cm, W: 58.5 cm, and H: 58.5 cm), two identical to-be-familiarized objects (cylindrical, D: 8 cm, H: 10 cm) were placed at the back left and right corners. The subject was placed at the midpoint of the wall opposite the objects in the apparatus, allowed to explore for 10 min, and returned to the home cage. After 1 h, one of the objects was replaced with a new object (cube, L: 10 cm, W: 10 cm, and H: 10 cm); the subject was placed back into the apparatus, being allowed to explore the two different objects for 10 min. The interaction time of exploring the two objects was recorded using an overhead video camera and analyzed using the EthoVision XT 8.5 software package (Noldus Information Technology; Leesburg, VA, United States).

#### Marble Burying

The marble-burying test is used for assessing the stereotyped and repetitive behavior relevant to ASD (Sharon et al., 2019). Marble burying was performed in a normal cage (L: 48 cm, W: 35 cm, and H: 20 cm) bottom with a floor area filled with 7–8 cm of fresh, autoclaved wood chip bedding (Sharon et al., 2019). The subject was habituated to the cage for 10 min, then returned to the home cage. Subsequently, the bedding was leveled, and 20 glass marbles (4 × 5) were placed on the top of the bedding. The subject was placed back into the cage and allowed to explore for 10 min. The number of buried marbles (50% or more covered) was recorded. The bedding was renewed for each subject, and marbles were cleaned with 75% ethanol.

### Tissue Collection, RNA, and Protein Extraction

On PND 42, juvenile rats from different groups were accepted anesthesia by first administering i.p. 10% chloral hydrate and then decapitated. Subsequently, brains were split using surgical scissors, and the brain region of Mpfc was collected into an RNase-free tube and kept frozen at −80°C until analysis. To investigate the effects of OT or AS-8351 administration in NMS rats, the brain tissue was collected after the final behavioral test of administration within 1 h on PND 42. Total RNA was extracted from mPFC tissue using RNA extraction reagent RNAiso Plus (Code No. 9109, TaKaRa BIO INC., Japan). Protein was extracted from PFC by radioimmunoprecipitation assay lysis buffer (Beyotime Biotechnology, China) with 1-mM phenylmethylsulfonyl fluoride and cocktail protease inhibitor (Roche, United States), boiled in 4× sodium dodecyl sulfate loading buffer for 5 min and then kept frozen in −20°C after being cooled to room temperature.

### Quantitative Real-Time Polymerase Chain Reaction

Quantitative real-time PCR was used to compare the mRNA levels. PrimeScript^TM^ RT reagent Kit with gDNA Eraser (TaKaRa BIO INC., Japan) was used to synthesize complementary DNA. Real-time PCR was performed using ChamQ Universal SYBR qPCR Master Mix (Vazyme Biotech, United States) and analyzed using LightCycler^®^ 480 SYBR Green Software II (Roche, United States). The executive programs of amplification were the following parameters: 95°C for 30 s, followed by 45 cycles of heating at 95°C for 10 s, annealing at 60°C for 30 s, and extension at 95°C for 15 s. Sequences of the primers were as follows: OXTR, forward ACCTGGATATGCGCAAGTGT, reverse GGGCAGGTAGTTCTCCTCCT; glyceraldehyde phosphate dehydrogenase (GAPDH), forward GACATGCCG CCTGGAGAAAC, reverse AGCCCAGGATGCCCTTTAGT. The relative expression of the respective genes was normalized to that of GAPDH within the same sample. ΔCt, indicating the difference between GAPDH and the target gene, was expressed by the formula ΔCt = Ct_*target gene*_ − Ct_*GAPDH*_, ΔΔCT was expressed by the formula ΔΔCT = ΔCT (treated group) − ΔCT (CONT group), and the relative expression of each mRNA level was calculated using the 2−^Δ^ ^Δ^ ^*Ct*^ method. The final value was represented as a value relative to the control group.

### Western Blotting

The protein samples from mPFC were separated on 12–8% sodium dodecyl sulfate-polyacrylamide gels and transferred to polyvinylidene fluoride filter (0.45 μm, Millipore, United States). The membrane was blocked by non-fat milk (5%) in Tris-buffered saline containing 0.1% Tween 20 and incubated with primary antibodies against including GAPDH (1:1,000, Affinity Biosciences, United States), OXTR (1:800, Abcam, United Kingdom), tri-methyl-histone H3 Lys4 (1:1,000, Cell Signaling Technology, United Kingdom), *p*-Erk1/2 (1:1,000, Cell Signaling Technology, United States), Erk1/2 (1:1,000, Cell Signaling Technology, United States), *p*-MEK1/2 (1:1,000, Cell Signaling Technology, United States), MEk1/2 (1:1,000, Cell Signaling Technology, United States), *p*-c-Raf (1:1,000, Cell Signaling Technology, United States), c-Raf (1:1,000, Cell Signaling Technology, United States), H3 (1:1,000, Cell Signaling Technology, United States), CREB (1:800, Cell Signaling Technology, United States), and *p*-CREB (1:800, Cell Signaling Technology, United States) overnight at 4°C. After 1-h incubation at 37°C with secondary antibodies including horseradish peroxidase (HRP)-conjugated rabbit anti-goat IgG (Affinity Biosciences, United States), HRP-conjugated goat anti-rabbit IgG (1:10,000, Absin, China), and HRP-conjugated goat anti-mouse IgG (Affinity Biosciences, United States) followed by visualization under iBright FL1000 imaging system (Thermo Fisher, United States) with chemiluminescence (ECL Advance; Amersham Biosciences). Protein band intensity was quantified using Image J software (National Institutes of Health, Bethesda, MD, United States), and the relative expression level of respective proteins was calculated by normalization to the GAPDH protein.

### Immunohistochemistry

Immunofluorescence staining was performed after behavioral tests. Rats were anesthetized with 10% chloral hydrate, followed by perfusion with 0.9% saline and 4% paraformaldehyde solutions. Brains were postfixed overnight in 4% paraformaldehyde at 4°C followed by dehydration with gradient sucrose in phosphate-buffered saline. Rat brain sections (30 μm) were blocked with 3% bovine serum albumin in 0.3% Triton X-100 for 1 h at room temperature and incubated for 24 h at 4°C with primary antibodies including OXTR (1:600, Abcam, United Kingdom), IBA-1 (1:500, Wako, Japan), NeuN (1:600, Cell Signaling Technology, United States), and glial fibrillary acidic protein (GFAP) (1:800, Proteintech, United States). After washing with phosphate-buffered saline three times for 30 min, the sections were incubated for 2 h at room temperature with secondary antibodies Alex Flour^®^ 594-conjugated goat anti-rabbit (1:600, Abcam, United Kingdom) and Alex Flour^®^ 488-conjugated donkey anti-goat (1:600, Absin, China). Expressions of OXTR, IBA-1, GFAP, and NeuN in mPFC were visualized under a laser scanning confocal microscope (Olympus, Japan). Nucleic staining reagent 4′,6-diamidino-2-phenylindole fluoromount-G (Southern Biotech, United States) was used to stain cell nuclei. Colocalization analysis was identified via utilizing ImageJ software (National Institutes of Health, Bethesda, MD, United States) that was equipped with a colocalization finder, under which colocalized pixels appeared white. Merged confocal images (20×) of neural cell markers (IBA-1, NeuN, GFAP, red) costained with OXTR (green) and 4′,6-diamidino-2-phenylindole (blue) in mPFC slices of CONT and long-term NMS rats were shown.

### Statistics

Data analyses were performed using GraphPad Prism 6 (GraphPad Software, Inc., La Jolla, CA, United States). For statistical significance, unpaired, two-tailed Student’s *t*-test was used to evaluate statistical significance between the two groups. One-way with Tukey’s *post hoc* tests or two-way analysis of variance (ANOVA) followed by *post hoc* Bonferroni tests were used to evaluate statistical significance in experiments with more than two groups. Two-way repeated measure ANOVA followed by *post hoc* Bonferroni tests was used to evaluate the weight loss of NMS. The level of statistical significance was set at *P* < 0.05. *F* values, degrees of freedom, and *P* values for all ANOVAs (representing statistics of *post hoc* comparisons and main interaction effects, unless otherwise stated), as well as degrees of freedom for *t*-tests and *t*-values, are shown in figures. Data were presented as mean ± SEM.

## Results

### Short-Term Neonatal Maternal Separation Stress Did Not Influence Social Recognition in Juvenile Rats

**FIGURE 1:**
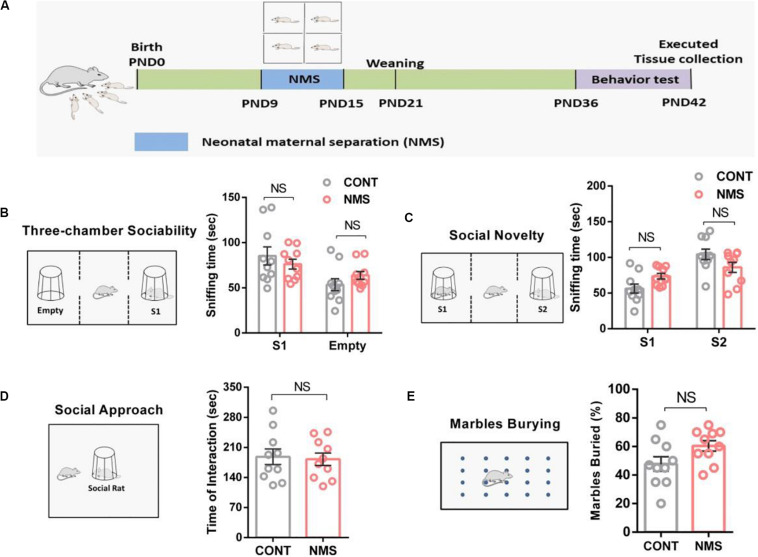
Juvenile rats suffering from short-term neonatal maternal separation did not display behavioral social deficits. **(A)** Experimental design: neonatal rats were subject to a short-term maternal separation from PND 9–15. **(B)** Three-chamber sociability test, plots showing the time spent investigating either the social (S1) or non-social (Empty) stimulus (two-way ANOVA followed by the *post hoc* Bonferroni’s multiple comparison tests, *F*_(1,36)_ = 1.975, *P* = 0.1685; n.s., *P* > 0.05). **(C)** Social novelty test, plots showing the time spent investigating either the familiar (S1) or unfamiliar (S2) social rat (two-way ANOVA followed by the *post hoc* Bonferroni’s multiple comparison tests, *F*_(1,36)_ = 1.821, *P* = 0.1425; n.s., *P* > 0.05). **(D)** Social approach test, plots showing the time spent investigating the social rat (two-tailed unpaired *t*-test, *t*_18_ = 0.4486, *P* = 0.6591, n.s., *P* > 0.05). **(E)** Marble-burying test, plots showing the percentage of buried marbles (two-tailed unpaired *t*-test, *t*_18_ = 0.596, *P* = 0.5973, n.s., *P* > 0.05). n.s., not significant. Data are represented as mean ± SEM (*n* = 10 in each group).

Early life stress confers lifelong stress susceptibility (Pena et al., 2017) and causes psychiatric diseases and cognitive impairment later in life, including social deficits relevant to ASD, anxiety, and depression (Chapman et al., 2004; Carr et al., 2013; Flouri et al., 2020). To examine the effects of ELS during the stress hyporesponsive period on social and cognitive–behavioral changes in juvenile rats, we used NMS as the ELS. The NMS offsprings were subjected to a daily 4 h maternal separation on PND 9–15 (Figure 1A), whereas the control littermates were kept with their mother without disturbances. After a short-term (a week) NMS stress in neonatal rats, behavioral tests were conducted on PND 36–42.

Here, the three-chamber test was used to assess cognitive changes induced by NMS in the form of general sociability and interest in social novelty, which was useful for quantifying deficits in social behavior in animals exhibiting autistic traits (Qin et al., 2018). Our behavioral results indicated that both the juvenile controls and the rats suffering from short-term NMS showed a similar preference for spending more time in the chamber containing a stimulus rat compared with the empty chamber (Figure 1B, two-way ANOVA followed by the *post hoc* Bonferroni’s tests, n.s. [not significant], *F*_(1,36)_ = 1.975, *P* = 0.1685; *p* > 0.05). We also used the social approach test to investigate the effect of short-term NMS on the sociability of juvenile rats. It showed that NMS rats spent similar time investigating the social rat (Figure 1D, two-tailed unpaired *t*-test, *t*_18_ = 0.4486, *P* = 0.6591; n.s., *p* > 0.05), suggesting that NMS rats did not show impairment in sociability. Likewise, no differences in social novelty were observed between the controls and NMS rats (Figure 1C, two-way ANOVA followed by the *post hoc* Bonferroni’s tests, *F*_(1,36)_ = 1.821, *P* = 0.1425; n.s., *p* > 0.05), which demonstrated that social novelty was not impaired in NMS rats. Furthermore, in the marble-burying test, NMS rats did not show repetitive and stereotypical behavior compared with the controls (Figure 1E, two-tailed unpaired *t*-test, n.s., *t*_18_ = 0.596, *P* = 0.5973; *p* > 0.05). These data discussed suggested that exposition to short-term NMS did not trigger social deficits and induced ASD-like behaviors in rats during juvenile.

### Long-Term Neonatal Maternal Separation Stress Showed Impacts on Social Recognition but Not Learning and Memory Functions

**FIGURE 2:**
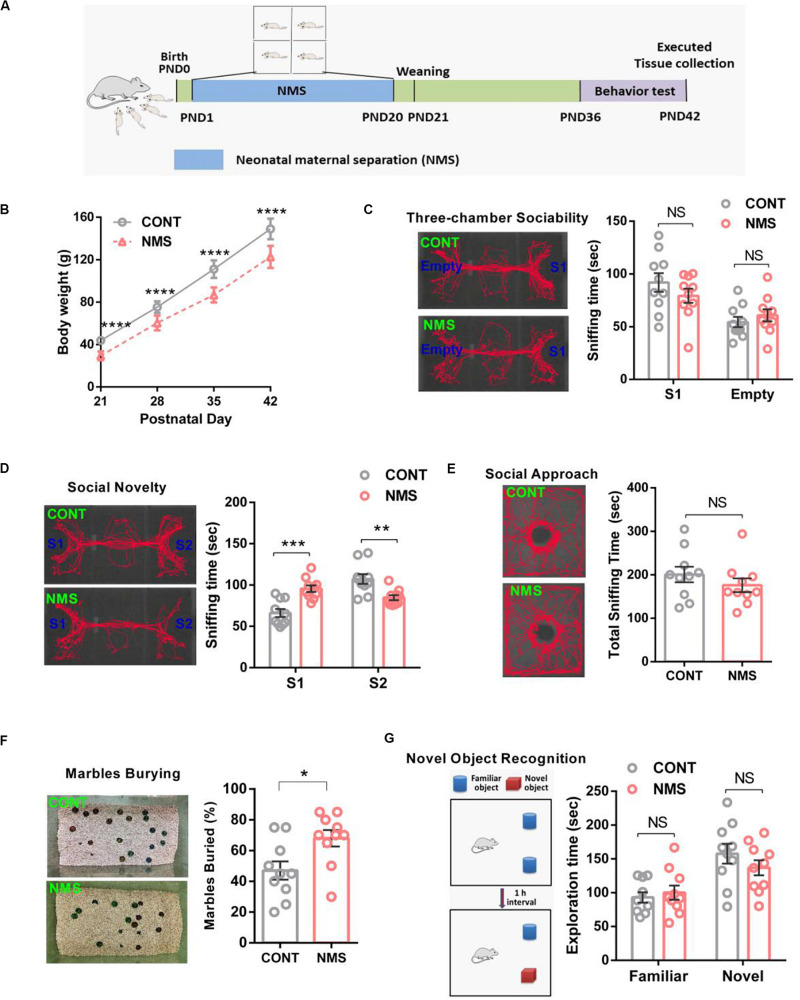
Effects of long-term neonatal maternal separation stress on bodyweight, social recognition, and memory recognition in juvenile rats. **(A)** Experimental design: neonatal rats were subject to a long-term maternal separation from PND 1–20. **(B)** Bodyweight at different time points (two-way repeated-measures ANOVA followed by the *post hoc* Bonferroni’s multiple comparison tests; *F*_(3,72)_ = 5.382, *P* < 0.0001, CONT vs NMS, ****P < 0.0001). **(C)** Three-chamber sociability test, plots showing the time spent investigating either the social (S1) or non-social (Empty) stimulus (two-way ANOVA followed by the *post hoc* Bonferroni’s multiple comparison tests; *F*_(1,36)_ = 2.013, *P* = 0.1645, NS, *P* > 0.05). **(D)** Social novelty test, plots showing the time spent investigating either the familiar (S1) or unfamiliar (S2) social rat (two-way ANOVA followed by the *post hoc* Bonferroni’s multiple comparison tests; *F*_(1,36)_ = 32.32, *P* < 0.0001, ***P < 0.001, **P < 0.01). **(E)** Social approach test, plots showing the time spent investigating the social rat (two-tailed unpaired *t*-test, *t*_18_ = 1.032, *P* = 0.3155, n.s., *P* > 0.05). **(F)** Marble-burying test, plots showing the percentages of buried marbles (two-tailed unpaired *t*-test, *t*_18_ = 2.634, *P* = 0.0168, *P < 0.05). **(G)** Novel object recognition test, plots showing the time spent investigating either the familiar or novel object (two-way ANOVA followed by the *post hoc* Bonferroni’s multiple comparison tests; *F*_(1,36)_ = 1.543, *P* = 0.2222, n.s., *P* > 0.05); n.s., not significant. Data are represented as mean ± SEM (*n* = 10 in each group).

As shown in Figure 1, short-term NMS did not affect the social cognition, including sociability and social novelty, and cause autistic-like behaviors; we thought that the different durations of stress in rodents might bring different behavioral outcomes. A previous study has revealed that the rats daily suffering from a maternal separation for 80 min on PND 2–14 showed more increased anxiety-like behaviors, susceptibilities to stress, and locomotor activities compared with the rats for 15 min on PND 2–14 (Lippmann et al., 2007). Hence, we further explored the effects of long-term NMS stress on cognitive functions in juvenile rats. For the long-term NMS procedure, the NMS offsprings were subjected to a daily 4-h maternal separation on PND 1–20 and then weaned on PND 21 (Figure 2A).

It is well known that weight loss is an independent risk and biomarker for mental illness and developmental impairment. During NMS, normal maternal lactation was interrupted. As a result, the bodyweight of long-term NMS rats was significantly lower than that of the control rats during PND 21–42 (Figure 2B, two-way repeated-measures ANOVA followed by the *post hoc* Bonferroni’s tests, *F*_(3,72)_ = 5.382, *P* < 0.0001). The behavioral results showed the rats experiencing long-term NMS still showed a similar preference for spending more time in the chamber containing a stimulus rat compared with the empty chamber (Figure 2C, two-way ANOVA followed by the *post hoc* Bonferroni’s tests, *F*_(1,36)_ = 2.013, *P* = 0.1645; n.s.), and the social approach test observed no differences in sociability between them (Figure 2E, two-tailed unpaired *t*-test, *t*_18_ = 1.032, *P* = 0.3155; n.s.), suggesting that long-term NMS did not have serious consequences on sociability. In the social novelty test, NMS rats spent less time in exploring the chamber containing a social rat in comparison with the controls, which suggested that NMS rats exhibited a lower preference for the stranger rat (Figure 2D, two-way ANOVA followed by the *post hoc* Bonferroni’s tests, *F*_(1,36)_ = 32.32, *P* < 0.0001). It has been reported that a lack of preference for social novelty is implicated in impairments in social recognition or memory, which are important early markers for ASD and neurodevelopmental illnesses with strong genetic susceptibility (Frazier and Goodwin, 2020). To further assess whether long-term NMS could cause repetitive and stereotypical behavior, a behavioral deficit involved in ASD, we performed the marble-burying test. The results showed that the juvenile rats subjected to long-term NMS exhibited repetitive and stereotypical autistic-like behavior compared with the control littermates (Figure 2F, two-tailed unpaired *t*-test, *t*_18_ = 2.634, *P* = 0.0168). In addition, in consideration that long-term NMS could lead to impairments in social recognition, we used novel object recognition to further investigate whether it affected the learning and recognition memory functions. As shown in the novel object recognition test, both the NMS rats and the controls displayed a similar preference for spending more time in the novel object than the familiar object (Figure 2G, two-way ANOVA followed by the *post hoc* Bonferroni’s tests, *F*_(1,36)_ = 1.543, *P* = 0.2222; n.s.), demonstrating that long-term NMS did not trigger deficits in learning and memory functions.

### Long-Term Neonatal Maternal Separation Induced Oxytocin Receptor Downregulation and Inhibition of Erk/MAPK Signaling in Medial Prefrontal Cortex

Experiences in early life have shown the potential to change social recognition and may lead to neuropsychiatric disorders, including ASD (Tabbaa et al., 2017; Brydges et al., 2019). As we found earlier, long-term repetitive NMS could cause impairments in social recognition and autistic-like behaviors. However, the underlying molecular mechanisms have still not been well understood. It has been found that early life experiences may influence behaviors via epigenetic-mediated mechanisms (Babenko et al., 2015). Recent studies have revealed that early rearing history affects OXTR epigenetic regulation in rhesus macaques and prairie voles, which may be involved in behavioral deficits later in life (Baker et al., 2017; Perkeybile et al., 2019).

**FIGURE 3:**
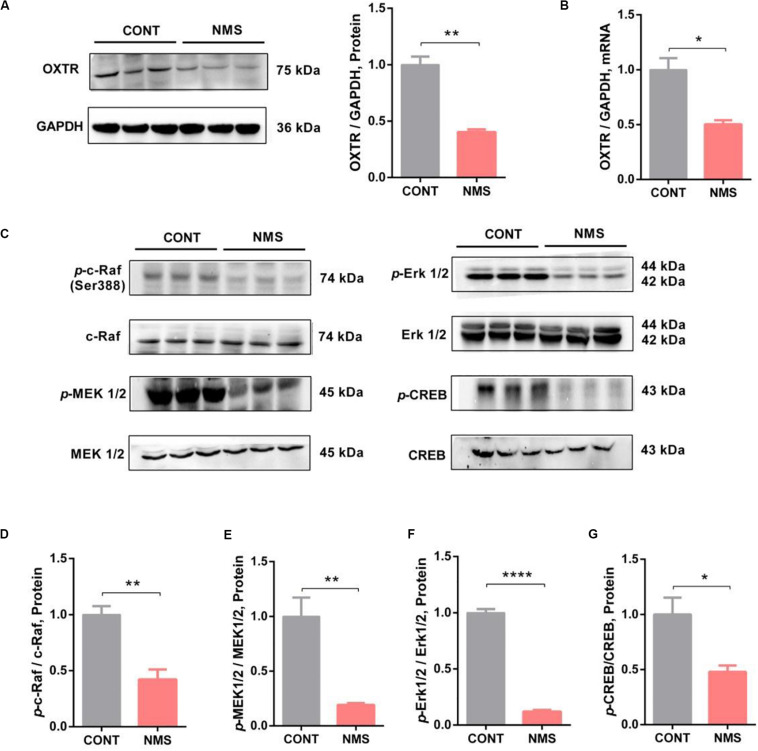
OXTR expression was downregulated with inhibition of Erk/MAPK signaling in long-term NMS rats. **(A)** Immunoblots and quantification analysis of the protein level of OXTR in mPFC from CONT and long-term NMS rats (two-tailed unpaired *t*-test, *t*_4_ = 7.869, *P* = 0.0014, **P < 0.01). **(B)** Quantitative real-time PCR data on mRNA level of OXTR in PFC from CONT and NMS rats (two-tailed unpaired *t*-test, *t*_4_ = 4.422, *P* = 0.0115, *P < 0.05). **(C–G)** Immunoblots and quantification analysis of the protein levels of *p*-c-Raf, *p*-MEK 1/2, *p*-Erk 1/2, and *p*-CREB in mPFC from CONT and long-term NMS rats (two-tailed unpaired *t*-test; *t*_4_ = 4.958 [*p*-c-Raf], *P* = 0.0077; *t*_4_ = 4.687 [*p*-MEK1/2], *P* = 0.0094; *t*_4_ = 25.46 [*p*-Erk1/2], *P* < 0.0001; *t*_4_ = 3.200 (*p*-CREB), *P* < 0.0329; **P < 0.01, ****P < 0.0001, *P < 0.05). Data are represented as mean ± SEM (*n* = 3 in each group).

Based on these recent findings, we hypothesized that OXTR might be implicated in long-term repetitive NMS-induced impairments in social recognition and autistic-like behaviors. Our data showed that OXTR gene expression in NMS juvenile rats was markedly downregulated in mPFC (Figure 3A, two-tailed unpaired *t*-test, *t*_4_ = 7.869, *P* = 0.0014), an important brain region that played an essential role in modulating social cognition, when compared with the controls littermates. The OXTR mRNA expression in mPFC was also dramatically decreased in NMS rats (Figure 3B, two-tailed unpaired *t*-test, *t*_4_ = 4.422, *P* = 0.0115). Consequently, our results indicated that long-term repetitive NMS could downregulate the protein and mRNA levels of OXTR in the mPFC of NMS rats.

OT, as the endogenous ligand of OXTR, exerting effects via OXTR, has been shown to improve social symptoms in ASD patients by intranasal administration due to its roles in regulating social behavior and improving anxiety (Yoshida et al., 2009; Yatawara et al., 2016; Parker et al., 2017). Rodents bearing knockout for either the OT gene or OXTR gene showed conspicuous social deficits (Modi and Young, 2012). As the expression of OXTR, in our findings, was downregulated in NMS rats, we then further investigated whether the downstream signaling of OXTR, namely, c-Raf/MEK/Erk/CREB pathway, was affected. Our results indicated that the phosphorylation of c-Raf/MEK/Erk/CREB signaling in mPFC was dramatically inhibited (Figures 3C–G, two-tailed unpaired *t*-test; *t*_4_ = 4.958 [*p*-c-Raf], *P* = 0.0077; *t*_4_ = 4.687 [*p*-MEK1/2], *P* = 0.0094; *t*_4_ = 25.46 [*p*-Erk1/2], *P* < 0.0001; *t*_4_ = 3.200 [*p*-CREB], *P* < 0.0329). Thus, the data suggested that long-term repetitive NMS could induce OXTR downregulation with inhibition of the downstream Erk/MAPK signaling.

### Oxytocin Treatment Improved Social Deficient Behavior in Juvenile Long-Term Neonatal Maternal Separation Rats Accompanied With Activation of Erk/MAPK Signaling

**FIGURE 4:**
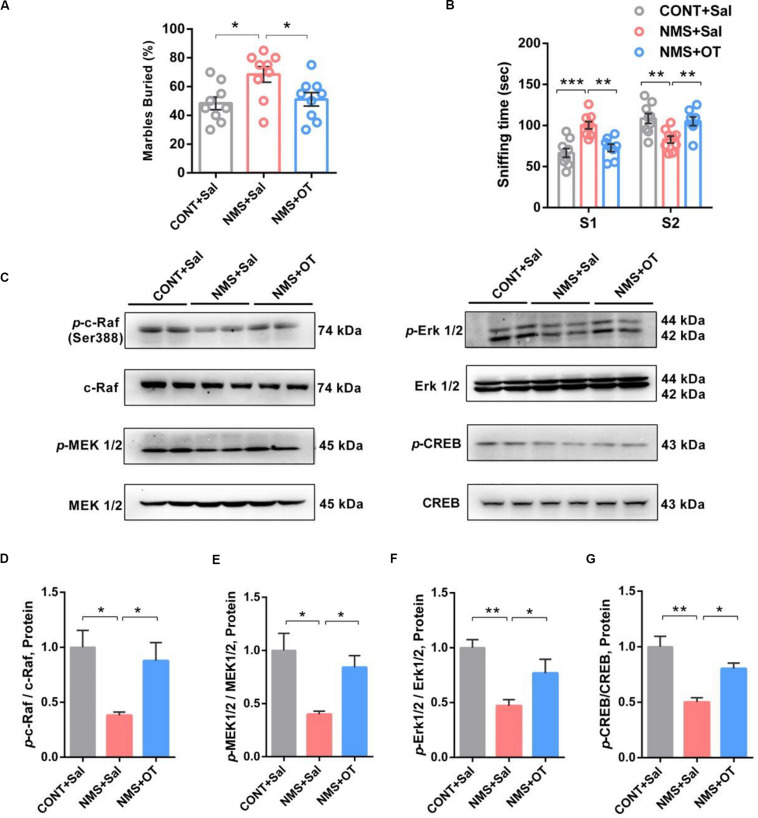
OT treatment rescued impairments in social recognition and autistic-like behaviors in juvenile long-term NMS rats accompanied with activation of Erk/MAPK signaling. **(A)** Marble-burying test, plots showing the percentages of buried marbles after OT treatment (one-way ANOVA followed by the *post hoc* Tukey’s tests; *F*_(2,24)Treatment_ = 4.983, *P* = 0.0155, *P < 0.05, *n* = 9 in each group). **(B)** Social novelty test, plots showing the time spent investigating either the familiar (S1) or unfamiliar (S2) social rat after OT treatment (two-way ANOVA followed by the *post hoc* Bonferroni’s multiple comparison tests; *F*_(2,46)_ = 20.50, *P* = 0.0003, ***P < 0.001, **P < 0.01, *n* = 9 in each group). **(C–G)** Immunoblots and quantification analysis of the protein levels of *p*-c-Raf, *p*-MEK 1/2, *p*-Erk 1/2, and *p*-CREB in mPFC from CONT + Sal., NMS + Sal., and NMS + OT rats after OT treatment (one-way ANOVA followed by the *post hoc* Tukey’s tests; *F*_(2,6)*Treatment*_ = 9.655 [*p*-c-Raf], *P* = 0.0133; *F*_(2,6)Treatment_ = 9.480 [*p*-MEK1/2], *P* = 0.0139; *F*_(2,6)Treatment_ = 15.18 [*p*-Erk1/2], *P* = 0.0045; *F*_(2,6)Treatment_ = 17.16 [*p*-CREB], *P* = 0.0033; **P < 0.01, *P < 0.05, *n* = 3 in each group). Data are represented as mean ± SEM.

Intranasal administration of OT is clinically used in patients with autism for improving social deficits (Modi and Young, 2012; Yatawara et al., 2016). Herein, we used OT to treat the juvenile rats subjected to NMS to examine its effects on NMS-induced autistic-like behaviors. It showed that OT administration was shown to decrease repetitive and stereotyped behavior in the marble-burying test (Figure 4A, one-way ANOVA followed by the *post hoc* Tukey’s tests, *F*_(2,24)Treatment_ = 4.983, *P* = 0.0155) and rescue the impairments in social recognition by increasing the preference for the strange rat in social novelty test (Figure 4B, one-way ANOVA followed by the *post hoc* Tukey’s tests, *F*_(2,46)_ = 20.50, *P* = 0.0003), indicating that OT treatment could improve NMS-induced social deficient and ASD-like behaviors in juvenile rat.

OT mediated by OXTR has been shown to improve social symptoms in autism, reduce anxiety, and induce long-term potentiation in the rodent brain through CREB phosphorylation via activation of c-Raf/MEK/ERK/CREB signaling (Blume et al., 2008; Yoshida et al., 2009). Western blotting results showed that OT treatment remarkably activated the c-Raf/MEK/Erk/CREB signaling in the mPFC of NMS rats via increasing phosphorylation of c-Raf/MEK/Erk/CREB pathway (Figures 4C–G, one-way ANOVA followed by the *post hoc* Tukey’s tests, *F*_(2,6)Treatment_ = 9.655 [*p*-c-Raf], *P* = 0.0133; *F*_(2,6)Treatment_ = 9.480 [*p*-MEK1/2], *P* = 0.0139; *F*_(2,6)Treatment_ = 15.18 [*p*-Erk1/2], *P* = 0.0045; *F*_(2,6)Treatment_ = 17.16 [*p*-CREB], *P* = 0.0033). Taken together, these data further supported the benefits of OT in improving social symptoms, accompanied by activation of Erk/MAPK cascade.

### Expression of Oxytocin Receptor in Neurons Was Decreased in Medial Prefrontal Cortex of Long-Term Neonatal Maternal Separation Rats

**FIGURE 5:**
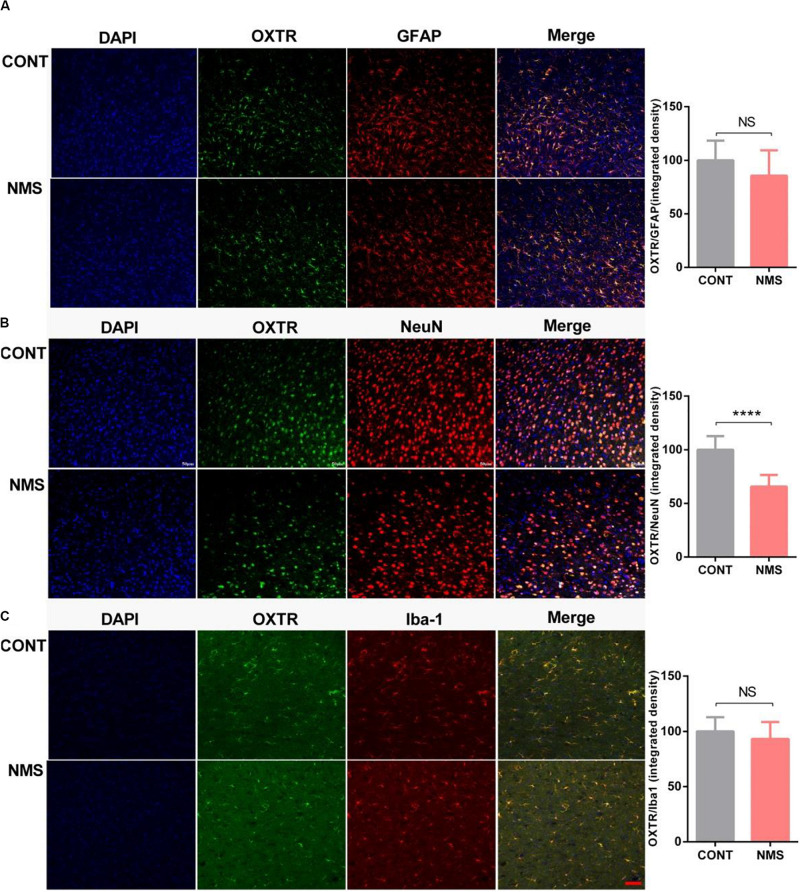
Expression of OXTR in neuronal cells, including neuron, microglia, and astrocyte in mPFC of long-term NMS rats. **(A–C)** Quantification of OXTR positive neural cells (integrated densities; NeuN for neuron marker; iBA1 for microglia marker; GFAP for astrocyte marker) in mPFC slices of different animal groups using immunofluorescence staining. Double immunolabeled surface areas of OXTR/GFAP **(A)**, OXTR/NeuN **(B)**, and OXTR/Iba-1 **(C)** from the mPFC, which are indicated in white lines, were quantified using the ImageJ Program (two-tailed unpaired *t*-test; *t*_22_ = 1.660 [OXTR/GFAP], *P* = 0.1110; *t*_22_ = 7.135 [OXTR/NeuN], *P* < 0.0001; *t*_22_ = 1.194 [OXTR/Iba-1], *P* = 0.2453; ****P < 0.0001, n.s. *P* > 0.05, *n* = 3 in each group). Data are represented as mean ± SEM. Scale bars: 50 μm.

Oxytocin receptor, a G protein-coupled receptor that is expressed on the cell membrane, is widely expressed in various tissues, especially in specific regions of the brain, including PFC, hippocampus, hypothalamus, and amygdala, mediating psychological behaviors (Busnelli and Chini, 2018). In neurons, the coupling of OTR with G protein transmits the signal intracellularly, permits OT to affect cell functions, and then causes the activations of intracellular signalings, such as Erk/MEK signaling, and PLC, the effector of phosphoinositide signaling system (Parent et al., 2008; Busnelli and Chini, 2018; Tirko et al., 2018). To further characterize the neuronal cells that express OXTR, we used immunohistochemistry to locate the coexpression of OXTR with different neuronal markers (NeuN for neuron, GFAP for astrocyte, and iBA-1 for microglia) in mPFC. As shown in Figures 5A–C, OXTRs were mainly expressed in neurons and astrocytes, a few in microglias. The integrated density of double immunostaining of OXTR/NeuN was significantly decreased in the mPFC of long-term MNS rats compared with control rats, whereas the integrated densities of OXTR in GFAP and Iba-1 were unchanged (two-tailed unpaired *t*-test; *t*_22_ = 1.660 [OXTR/GFAP], *P* = 0.1110; *t*_22_ = 7.135 [OXTR/NeuN], *P* < 0.0001; *t*_22_ = 1.194 [OXTR/Iba-1], *P* = 0.2453).

### Low Level of Histone Methylation of Tri-Methylation of Lysine 4 on Histone H3 in Medial Prefrontal Cortex of Long-Term Neonatal Maternal Separation Rats

**FIGURE 6:**
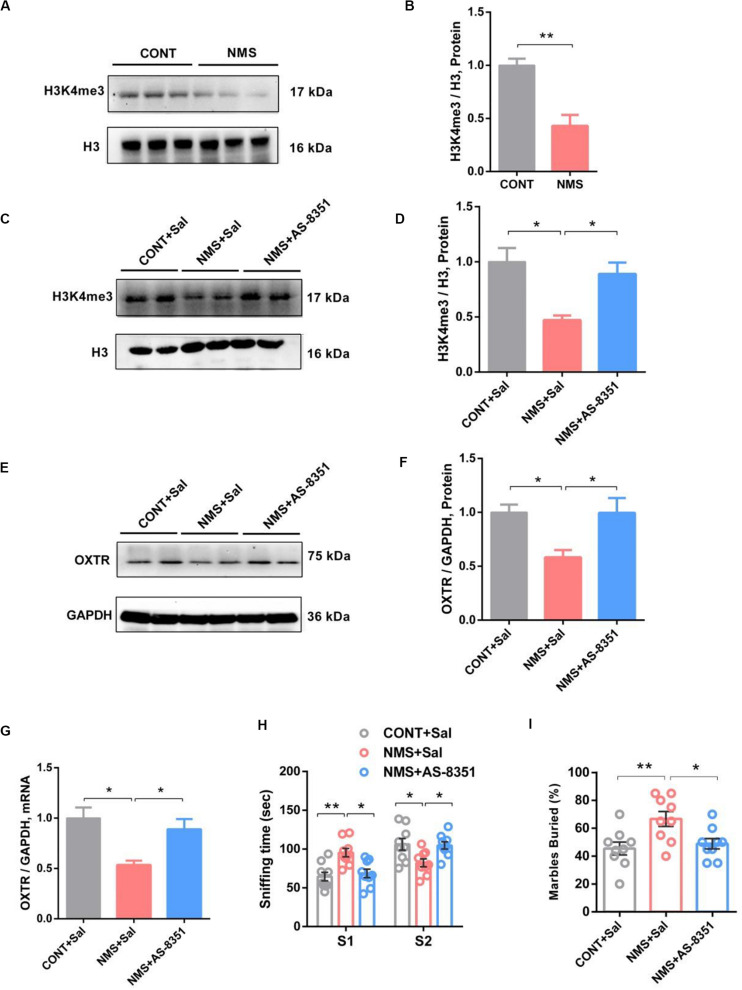
HDM inhibitor treatment restored NMS-induced social deficits with upregulation of H3K4me3 and OXTR in mPFC of long-term NMS rats. **(A,B)** Immunoblots and quantification analysis of the protein levels of H3K4me3 in mPFC from CONT and long-term NMS rats (two-tailed unpaired *t*-test, *t*_4_ = 4.738, *P* = 0.0091, **P < 0.01, *n* = 3 in each group).). **(C–F)** Immunoblots and quantification analysis of the protein levels of H3K4me3 and OXTR in mPFC from CONT + Sal., NMS + Sal., and NMS + AS-8351 rats after AS-8351 treatment (one-way ANOVA followed by the *post hoc* Tukey’s tests; *F*_(2,6)Treatment_ = 5.963 [H3K4me3], *P* = 0.0375; *F*_(2,6)Treatment_ = 11.04 (OXTR), *P* = 0.0098; *P < 0.05, *n* = 3 in each group). **(G)** Quantitative PCR showing OXTR mRNA and level in mPFC from CONT + Sal., NMS + Sal., and NMS + AS-8351 rats after AS-8351 treatment (one-way ANOVA followed by the *post hoc* Tukey’s tests; *F*_(2,6)Treatment_ = 10.87, *P* = 0.0101; *P < 0.05, *n* = 3 in each group). **(H)** Social novelty test, plots showing the time spent investigating either the familiar (S1) or unfamiliar (S2) social rat after AS-8351 treatment (two-way ANOVA followed by the *post hoc* Bonferroni’s multiple comparison tests; *F*_(2,48)_ = 14.09, *P* < 0.0001; **P < 0.01, *P < 0.05, *n* = 9 in each group). **(I)** Marble-burying test, plots showing the percentages of buried marbles after AS-8351 treatment (one-way ANOVA followed by the *post hoc* Tukey’s tests; *F*_(2,24)Treatment_ = 4.983, *P* = 0.0155; **P < 0.01, *P < 0.05, *n* = 9 in each group). Data are represented as mean ± SEM.

Recent findings have shown that experiences in early life can epigenetically tune the expression of OXTR, which has been associated with psychiatric disorders and individual differences, including impairments in social recognition and autistic-like behaviors in neural response to social stimuli (Perkeybile et al., 2019). Epigenetic regulatory alteration of OXTR was involved in biological mechanisms underlying the aberrant social behaviors (Tops et al., 2019). Similar findings have indicated that early maternal deprivation in rhesus macaque leads to significantly decreased H3K4me3 (an epigenetic marker for transcriptional activation) binding at OXTR (Baker et al., 2017). Thus, we examined the H3K4me3 level in the mPFC of NMS rats. Our data showed that the H3K4me3 level in NMS rats was markedly downregulated in comparison with the control littermates (Figures 6A,B, two-tailed unpaired *t*-test, *t*_4_ = 4.738, *P* = 0.0091).

### Histone Demethylases Inhibitor As-8351 Treatment Increased Histone H3K4 Tri-Methylation and Oxytocin Receptor Expression in Medial Prefrontal Cortex of Neonatal Maternal Separation Juvenile Rats and Restored Long-Term Neonatal Maternal Separation-Induced Social Deficits

Early nurture showed the ability to epigenetically tune the expression of OXTR by regulation of the H3K4me3 level (Tops et al., 2019). H3K4me3, serving as an epigenetic marker for transcriptional activation of genes, could provide support for the increase of the OXTR mRNA level, which was regulated by HDMs and HMTs (Greer and Shi, 2012; Miller, 2016; Baker et al., 2017). It was found that inhibition of KDM5B, a critical HDM, could increase OXTR expression by upregulating the H3K4me3 level (Miller, 2016). Herein, to further investigate whether inhibition of HDMs could change NMS stress-induced OXTR expression through epigenetic modulation of histone methylation pathway and then alleviate the impairments in social recognition and autistic-like behaviors, we used a selective KDM5B inhibitor of AS-8351 (Zhang et al., 2019) to treat NMS rats on PND 21–35.

Our studies showed that treatment with AS-8351 could significantly increase the histone methylation of H3K4me3 level (Figures 6C,D, one-way ANOVA followed by the *post hoc* Tukey’s tests, *F*_(2,6)Treatment_ = 5.963, *P* = 0.0375) and upregulate the OXTR expression both in protein synthesis and mRNA transcription (Figures 6E–G, one-way ANOVA followed by the *post hoc* Tukey’s tests, *F*_(2,6)Treatment_ = 11.04 [protein], *P* = 0.0098; *F*_(2,6)Treatment_ = 10.879 [mRNA], *P* = 0.0101) in the mPFC of long-term NMS rats. Behavioral results suggested that the NMS treated with AS-8351 showed an increased preference for the stranger rat in the social novelty test (Figure 6H, two-way ANOVA followed by the *post hoc* Bonferroni’s tests, *F*_(2,48)_ = 14.09, *P* < 0.0001) and decreased repetitive and stereotyped behavior in the marble-burying test (Figure 6I, one-way ANOVA followed by the *post hoc* Tukey’s tests, *F*_(2,24)Treatment_ = 4.983, *P* = 0.0155) in comparison with NMS rats. These data indicated administration with the HDM inhibitor rescued the social deficit behaviors observed in NMS subjects via restoring the expression of OXTR.

## Discussion

As early life is increasingly recognized as a critical period for brain development and neural plasticity, ELSs during the stage, especially in an infant, may exert long-lasting adverse influences on the brain functions and cause an individual susceptible to psychiatric disorders later in subsequent adolescence and adulthood, including anxiety, depression, and impairments in social recognition implicated in ASD (Bevins and Besheer, 2006; Liu et al., 2017). As neonatal stress of disruption in mother–infant contact, NMS results in multiple behavioral changes in rodents, such as depression, anxiety, and visceral hypersensitivity, and increases the risk of disease later in life (Carlyle et al., 2012; Seo et al., 2016; Loewy et al., 2019). In our study, we found that short-term NMS had no effects on general sociability, interest in social novelty, and stereotypical behavior, whereas long-term repetitive NMS could lead to impairments in social recognition and autistic-like behaviors by decreasing the preference for the stranger rat in social novelty and increasing stereotypical behavior in the marble-burying test compared with the littermates, without affecting the learning and memory functions. Furthermore, it did not exclude the possibility that the starting time of stress might also affect behavioral phenotypes. The early postnatal stage, specifically PND 4 to PND 14 in rats, is crucial for the development of the stress system and is considered as the stress hyporesponsive period (Schmidt et al., 2003). As the two paradigms started at different PND, this brought about an assumption whether another short-term NMS (starting from PND 1 to 7) would cause impairments in behaviors observed in the long-term NMS (PND 1–20), which was indeed a limitation of this study. Thus, it might be concluded that the duration, severity of stress, and starting time of stress might be implicated in the behavioral phenotypes.

It was found that long-term repetitive NMS caused significant weight loss, which might also trigger behavioral changes in particular when the animals were under a subinflammatory state (Rideau Batista Novais et al., 2016; Mairesse et al., 2019). Maternal deprivation was severe psychosomatic stress that could induce many abnormal changes besides weight loss. Even the NMS rats showed significant weight loss during adolescence; they also displayed other normal behaviors, including memory recognition and sociability, except for impairments in social novelty and stereotyped behavior. We considered that weight loss was part of the consequences that resulted from maternal deprivation due to the neonatal stress, which might not be the primary cause of the behavioral deficits but an inevitable result of neonatal maternal deprivation that led to a nutritional deficiency during the stress.

Exposure to NMS, serving as an early life adverse event, showed the potentiality of increasing individual vulnerability to later psychiatric disorders in subsequent life and led to impairments in social recognition and autistic-like behaviors (Kambali et al., 2019). Our findings also showed that long-term repetitive NMS induced deficits in the development of the social system. However, the underlying molecular mechanism has not largely been elucidated. Related studies suggested that early experiences, including the quality and extent of maternal care, exerted long-lasting alterations in social behaviors, anxiety, and fearfulness, along with aberrant HPA axis activity and the expression of brain-derived neurotrophic factor (Levine, 1970; Meaney, 2001; Daniels et al., 2004; Seo et al., 2016; Liu et al., 2017). Early rearing history had an effect on OXTR expression (Baker et al., 2017), which was consistent with our findings, indicating that disruption in maternal care in rats resulted in remarkable downregulation of OXTR repression in mPFC, a key brain region that played an essential role in mediating social behavior (Amodio and Frith, 2006). OXTR is widely expressed in different functional brain regions, including PFC, hippocampus, amygdala, and hypothalamus, exerting important roles in the regulation of complex social behaviors, as well as in psychiatric disorders characterized by social deficits (Bakermans-Kranenburg and van Ijzendoorn, 2014). OT is released in the paraventricular nucleus and supraoptic nucleus of the hypothalamus in the brain and also implicated in regulating the social behaviors besides mating, maternal behavior, attachment, aggression, and sexual behavior. Recent studies have demonstrated that OT produces anxiolytic, antidepressant, and pro-social effects in animal models (Ferguson et al., 2001; Heinrichs et al., 2003; Sharma et al., 2019). Due to its functions on the improvement of social recognition, the intranasal administration of OT has been under clinical studies for the treatment of ASD characterized by social deficits in adults and juveniles (Anagnostou et al., 2012). Studies also demonstrated that OXTR variants were involved in children subjected to emotional neglect, and deficits in OXTR or OT expression could result in pervasive social deficits in mice (Takayanagi et al., 2005; Womersley et al., 2019). OT/OXTR system is crucial in the regulation of social behaviors; as a result, NMS-induced OXTR downregulation may be implicated in the impairments of social recognition and autistic-like behaviors. The actions of both central and peripheral OTs are mediated through OXTR. As a G protein-coupled receptor that can receive and then transduce information into cells via signaling pathways, OXTR is expressed on the neural cell membrane in the brain, such as neuron and astrocyte (Hidema et al., 2016). The integrated density of OXTR on neurons by double immunostaining was significantly reduced, which might affect the functions of neurons. The main signaling pathways activated by the OXTR in neuronal cells are closely related to the intracellular effects and contribute to the qualitative and quantitative outcomes of OT responses in the brain. It has been reported that c-Raf/MEK/Erk/CREB (Erk-MAPK) signaling cascade functions as the intracellular downstream pathway of OT/OXTR (Tomizawa et al., 2003). Moreover, Neumann et al. (2013) demonstrated that OXTR/Erk/MAPK signaling mediated OT anxiolytic effects via activating the phosphorylation (Blume et al., 2008; Jurek et al., 2012; van den Burg et al., 2015). Besides the downregulation of OXTR, our data showed that the OXTR downstream signaling of Erk/MAPK cascade was remarkably inhibited in the PFC of NMS rats, which might be involved the downregulation of genes and the ELS-induced impairments of behaviors or the reactiveness in the response of stresses. OT has quite a limited ability to penetrate the blood–brain barrier and can only rely on related receptor transport (Leng and Ludwig, 2016; Duarte-Guterman et al., 2020), which is the reason that the current clinical administration is mainly through intranasal administration. In contrast, it has been reported that peripheral OT administration improves not only depressive-like behavior but also object recognition and social recognition in high fat diet-fed mice and can increase OT mRNA expression in the hypothalamus OT (Hayashi et al., 2020). Moreover, peripheral delivery of OT could lead to the central release of endogenous OT (Neumann et al., 2013). After i.p. injection of OT, NMS rats showed improved social deficient behavior accompanied by activation of Erk/MAPK signaling, indicating that inhibition of OXTR/Erk/MAPK signaling might be involved in NMS-induced social impairments. Furthermore, there were papers reporting that epigenetic variations, including DNA methylation, histone methylation, and acetylation, were involved in environmental exposure and psychiatric disease burden resulting from persistent disruptions of mother–infant interactions (McGowan et al., 2009; Tops et al., 2019). Early life adverse experiences such as childhood abuse influenced HPA function and led to aberrant behaviors in rats through epigenetic programming of gene expression (McGowan et al., 2009). A similar study showed that disruptions of mother–infant interactions in rhesus macaques resulted in a dramatic decrease of OXTR mRNA via epigenetic regulation of decreased H3K4me3, which contributed to the behavioral pathology observed in rhesus macaques that are subjected to early maternal deprivation (Baker et al., 2017). Epigenetic variability in the human OXTR gene caused by environmental events, especially those occurring during early childhood, was associated with the alteration of social sensitivity (Kraaijenvanger et al., 2019). In the prairie vole, early nurture could epigenetically tune the OXTR, which was associated with individual differences in neural response to social stimuli and impairments in social behavior (Perkeybile et al., 2019). Recently, it was reported that maternal deprivation in rhesus macaque caused alterations of H3K4me3 level binding at genes using a ChIP-sequencing assay and that the most robust gene is OXTR, which was correlated with the phenotypes of separation anxiety and arousal observed (Baker et al., 2017). NMS rats showed an epigenetic down-modulation of OXTR with a decreased H3K4me3 level in mPFC. H3K4me3, a marker for transcriptional activation, could provide support for the increase of the OXTR mRNA transcriptional level by facilitating the transcription factor to bind to the OXTR promoter (Mackowiak et al., 2014; Dincer et al., 2015; Baker et al., 2017; Varma et al., 2017). H3K4me3 level is mediated by HDMs and HMTs (Kouzarides, 2007). Inhibition of HDMs, which serve as a main regulatory factor of H3K4me3, could increase OXTR expression via upregulating H3K4me3 (Miller, 2016). Our results showed that NMS rats administrated with AS-8351, a potent inhibitor of HDMs, presented an increase of H3K4me3 OXTR levels in mPFC and restored social behaviors. Those findings indicated that disruptions of mother–infant interactions had an epigenetic effect on OXTR expression via regulation of H3K4me3, which involved NMS-induced impairments in social recognition and autistic-like behaviors.

## Conclusion

Early life stresses, especially during the neonatal or infant period, significantly increase the susceptibility of mental illness in juveniles and adult individuals and may lead to impairments in social recognition and autistic-like behaviors in subsequent life. Here, NMS in rats was used as a stress model of disruption in mother–infant contact. Our behavioral results showed that juvenile rats subjected to short-term NMS did not induce autistic-like behavioral deficits in social novelty, social approach, and marble-burying tests. In contrast, long-term NMS stress (PND 1–20) triggered impairments in social recognition or memory repetitive and stereotypical autistic-like behavior in juvenile MNS rats compared with the control littermates without affecting the learning and memory functions in the novel object recognition test. The molecular mechanism study underlying the NMS-induced social deficits showed that OXTR expression in the mPFC of NMS rats was evidently downregulated, especially in neurons, along with the inhibition of its downstream signaling of the Erk/MAPK cascade. OT treatment could rescue NMS-induced behavioral deficits with phosphorylation activation of the Erk/MAPK cascade. Moreover, our findings showed that NMS rats exhibited a low level of H3K4me3, which is involved in promoting the expression of OXTR. Treatment with the inhibitor of H3K4 demethylase alleviated the abnormal behaviors in NMS rats and increased the expression of OXTR in mPFC. Those findings identified a mechanism by which repetitive long-term disruption in mother–infant contact influenced later displays of social and autistic-like behaviors and suggested an epigenetic tuning of OXTR for NMS stress.

## Data Availability Statement

The data from the present study are available from the corresponding author on reasonable request.

## Ethics Statement

The animal study was reviewed and approved by Experimental Animal Committee of Shanghai Jiao Tong University School of Medicine.

## Author Contributions

JC, JW, and Y-XW conceived and designed the experiments. JW, LM, BY, and PJ performed the experiments. JC and JW analyzed the data. JW, JC, and Y-XW preparation of the manuscript. All authors contributed to the article and approved the submitted version.

## Conflict of Interest

The authors declare that the research was conducted in the absence of any commercial or financial relationships that could be construed as a potential conflict of interest.

## Abbreviations

ASD: Autism spectrum disorder
ELS: early life stress
H3K4me3: tri-methylation of lysine 4 on histone H3
HDMs: histone demethylases
HMTs: methyltransferases
mPFC: medial prefrontal cortex
NMS: neonatal maternal separation
OT: oxytocin
OXTR: oxytocin receptor.
